# Comparative genomics of resistance of spruce to the white pine weevil in British Columbia

**DOI:** 10.1186/1753-6561-5-S7-O10

**Published:** 2011-09-13

**Authors:** Kermit Ritland, Sébastien Verne, Barry Jaquish, Carol Ritland

**Affiliations:** 1 University of British Columbia, USA; 2 Ministry of Forests, Lands and Natural Resource Operations, USA

## 

We present results of two large scale comparative studies of the genomic basis of resistance of Interior spruce to the white pine weevil. Both volume growth, the main objective of the spruce breeding program in British Columbia, and white pine weevil resistance, are examined. "Interior spruce" is a species complex involving mainly *Picea glauca* (White spruce) but introgressed with *P. engelmannii* (Englemann spruce), depending upon locality. In the first study, we compared constitutive expression of 17825 genes between 20 resistant and 20 susceptible trees to the weevil; 54 upregulated and 137 downregulated genes were found in resistant phenotypes, with implications discussed in regard to volume growth. In particular, we will be surveying these genes for SNPs that differ between these two classes of trees in the next year. In the second study, we developed a 1536 Illumina SNP chip based upon candidate genes for weevil resistance. In a novel experimental design, we assayed 945 open-pollinated progeny of the Prince George breeding population (176 parents), and 654 open-pollinated progeny of the Prince Rupert breeding population (134 parents); parents were also genotyped. Within each family of 100 progeny, we identified the highest ranked 3 progeny and the lowest ranked 3 progeny, based upon BC Ministry of Forests scores for volume growth and resistance. These were genotyped and used in a novel test analogous to the transmission disequilibrium test to detect both SNP associations and QTLs linked to SNP markers. Discoveries about associations and QTL are discussed, with the added caution about genotyping error. Both studies illustrate how operational tree breeding populations can provide valuable inferences about tree genomics.

